# Effect of the Storage Conditions and Freezing Speed on the Color and Chlorophyll Profile of Premium Extra Virgin Olive Oils

**DOI:** 10.3390/foods12010222

**Published:** 2023-01-03

**Authors:** Anna Díez-Betriu, Julen Bustamante, Agustí Romero, Antonia Ninot, Alba Tres, Stefania Vichi, Francesc Guardiola

**Affiliations:** 1 Departament de Nutrició, Ciències de l’Alimentació i Gastronomia, Campus de l’Alimentació de Torribera, Facultat de Farmàcia i Ciències de l’Alimentació, Universitat de Barcelona, 08921 Santa Coloma de Gramenet, Spain; 2 Institut de Recerca en Nutrició i Seguretat Alimentària (INSA-UB), Universitat de Barcelona (UB), 08921 Santa Coloma de Gramenet, Spain; 3 Institute of Agrifood Research and Technology (IRTA), Mas Bové Ctra. Reus-El Morell km 3.8, 43120 Constantí, Spain

**Keywords:** premium EVOO, storage conditions, freezing speed, color, chlorophylls, pheophytinization

## Abstract

Premium extra virgin olive oils (PEVOO) are oils of exceptional quality and retail at high prices. The green color of recently extracted olive oils is lost during storage at room temperature, mainly because of the pheophytinization of chlorophylls. Since a green color is perceived as a mark of high-quality oils by consumers, it is especially important for PEVOO to maintain their initial green color. This study assessed the effect of applying low temperatures (refrigeration and freezing) and modified atmospheres on the color of four PEVOO for 24 months. Also, the effect of two freezing methods (slow freezing by placing the oil at −20 °C and fast freezing by immersing the oil in a bath of liquid nitrogen) was studied. Results showed that the green color was better preserved in oils frozen and stored at −20 °C whereas in oils frozen with liquid nitrogen the green color was lost much faster during frozen storage. An in-depth study of this unexpected phenomenon showed that this loss of green color was mainly due to a pheophytinization of chlorophylls. This phenomenon did not happen at the moment of freezing with liquid nitrogen, but over the first 100 days of storage at −20 °C. In addition, correlations between single chlorophyll and pheophytin contents and chromatic coordinates were established.

## 1. Introduction

Extra virgin olive oil (EVOO) is a highly priced vegetable fat due to its great nutritional and sensory value, as well as its health benefits. In recent years, advances in the scientific knowledge and improvement of production techniques have led to an increase in the quality of virgin olive oils. As a consequence, 20% of the EVOO on the market are nowadays being labelled as “premium” [1]. Although this is not an official commercial category and premium oils are included in the “extra virgin” group, various initiatives promoted by EVOO producers, such as the Extra Virgin Alliance (EVA), the Ultra Premium Extra Virgin Olive Oil (UP) certification and the 3E Association (Ethics, Excellence, Economy) propose good practice guides and more restrictive quality standards to differentiate Premium extra virgin olive oils (PEVOO) in the market [2,3,4]. These PEVOO are oils of exceptional quality that retail at high prices [5] due to the outstanding characteristics that distinguish them from other standard EVOO.

It is well known that EVOO experiences a decrease in its quality during storage, although it does not normally lead to a decline in its commercial category between harvests. However, in the case of PEVOO, this decrease of quality could be enough to cause the loss of its distinctive quality features, which would compromise its availability to the first months after harvest. The control of the storage conditions is fundamental to maintain the quality of PEVOO [6] and therefore improve its commercialization. The higher price that consumers are willing to pay for oils with outstanding quality features compared to standard EVOO [7] would justify higher storage costs. For this reason, the benefits of applying conditions that are not common in the olive oil sector, such as controlled atmosphere and freezing storage, deserve to be evaluated.

Consumers willing to pay high prices for PEVOO have a special interest in their quality features, including color [7]. In this regard, several acceptability studies have shown that green color of EVOO is among the attributes associated with high-quality oils by consumers [5,7,8,9], who perceive it as an assurance of high-quality level [8]. In fact, color of the oil proved to be more relevant than its origin in the formation of consumer preferences [10].

The green color of olive oil is due to chlorophyll pigments [11], which comprise bluish-green chlorophyll *a* and yellowish-green chlorophyll *b*, and whose composition mainly depends on the olive fruit cultivar and ripening degree [12]. However, during fruit milling and paste beating, pheophytins *a* and *b* start to form when the magnesium ion in the chlorophyll molecules is replaced by two hydrogen atoms, mainly as a consequence of a pH decrease [11,13]. This process, called pheophytinization, involves a shift of color from greenish to brownish hues. The chlorophyll profile in fresh virgin olive oil is thus determined by the *a* and *b* forms initially present in the fruits and their evolution to pheophytin derivatives during oil extraction [13].

During oil storage, the chlorophyll and pheophytin profile may undergo further modifications. The pheophytinization of chlorophylls that had started during oil extraction advances throughout oil storage, especially in the presence of light, causing a drastic decrease of chlorophylls [14,15]. Although to a lesser extent, this phenomenon has also been reported to take place during oil storage in the dark [13,16]. In particular, pheophytinization of chlorophyll *a* is faster than chlorophyll *b*, being complete after 4 months of storage in the dark between 15 and 19 °C [13,16]. Consequently, the green color of recently extracted virgin olive oil is progressively lost under conventional storage conditions. Chlorophylls and pheophytins may undergo oxidative degradation, generating allomerized forms [13]. Several studies have suggested oxygen availability to be a critical factor for their formation [13,17,18]. Moreover, as they act as photosensitizers, in the presence of light they accelerate the oil oxidation via singlet oxygen production [19,20].

The degradation of chlorophyll pigments is especially critical for the above mentioned PEVOO, since it implies a loss of green color intensity, and it negatively impacts their distinctive color hues. A proper control of the storage conditions may be crucial to maintain longer the color features of PEVOO. On the one hand, since temperature influences the speed of reactions, it could be expected that pheophytinization takes place at a slower pace when storing the oils at low temperatures, thus maintaining the green color for a longer period of time. To date, only one study has evaluated the effect of low storage temperatures on the content of total chlorophylls of EVOO, although its effect on the color was not reported [21]. On the other hand, oxygen availability could play a role in the oxidation of chlorophylls and pheophytins during olive oil storage [13,18,22,23], which would in turn affect its color. The high value of premium EVOO could justify the cost entailed by the application of cold storage, yet more research on the preservation of the sensory quality at freezing temperatures is needed. The aim of the present study was to evaluate the effect of oxygen availability and storage temperature (room temperature, 4 °C and −20 °C) on the color of PEVOO. As a higher oxidation rate than the expected has been observed during the freezing phase transition of oils [24], the effect of two different freezing speeds followed by storage at −20 °C on the color of PEVOO was also assessed. Furthermore, the chlorophyll and pheophytin profile was studied in detail in the frozen PEVOO.

## 2. Materials and Methods

### 2.1. Experimental Designs

#### 2.1.1. Color Evaluation during PEVOO Storage under Different Conditions and Its Correlation with Chlorophyll and Pheophytin Content (Study I)

Four filtered PEVOO produced during the 2016/17 campaign, two of the ‘Arbequina’ cultivar and two of the ‘Picual’ cultivar, were obtained from La Gramanosa (Avinyonet del Penedès, Barcelona, Spain) and Castillo de Canena (Canena, Jaén, Spain), respectively. For each cultivar, one sample was produced at the beginning and one at the end of the harvest.

After homogenization, aliquot samples (100 mL) of each oil were placed in 130 mL borosilicate glass 3.3 bottles (23% headspace) with high density polypropylene caps from Scharlau (Sentmenat, Spain) and stored in the dark for 24 months. Half of the samples were submitted to a nitrogen stream before being closed, obtaining a low oxygen headspace (N), while the headspace of the other half contained air (O). The samples were stored at three different temperatures: 20–25 °C (room temperature, RT), 4 °C (refrigerated, R) and −20 °C. Two methods were used in order to freeze the samples stored at −20 °C: a slow freezing method (S), by putting the samples directly in the freezer at −20 °C, and a fast-freezing method (F), by immersing the samples in a bath of liquid nitrogen.

One aliquot for each oil, storage condition and point of analysis was prepared. Color measurements were performed in duplicate for each sample and condition at the beginning of the experiment and at 6, 12 and 24 months. Chlorophyll and pheophytin content were measured in duplicate in the frozen samples (S and F) at 12 and 24 months. Altogether, 100 samples were analyzed for color, that is, 4 at time 0 and 96 during the conservation study: 4 oils × 2 headspace compositions (O, N) × 4 storage temperatures and freezing methods (RT, R, S and F) × 3 storage times (6, 12 and 24); while 32 samples were analyzed for chlorophyllic pigments (4 oils × 2 headspace compositions (O, N) × 2 freezing methods (S, F) × 2 storage times (12, 24)) (Table 1).

#### 2.1.2. Chlorophyll Pheophytinization in Fast-Frozen PEVOO (Studies II, III and IV)

##### Monitoring of Color and Chlorophyll Profile at 0, 6, 12 and 24 Months of Storage at −20 °C after Fast and Slow Freezing (Study II)

One filtered PEVOO of the ‘Picual’ cultivar was obtained during the 2017/18 harvest from Castillo de Canena (Canena, Jaén, Spain). Sample aliquots of 100 mL (see Section 2.1.1.) were frozen using the two methods explained in Section 2.1.1. and were stored at −20 °C in the dark for 24 months. Chlorophyll and pheophytin content, as well as color, were measured in duplicate for each sample and condition at the beginning of the experiment and at 6, 12 and 24 months (Table 1).

##### Analysis of Chlorophyll Profile after Fast and Slow Freezing and 24 h of Storage at −20 °C (Study III)

One filtered PEVOO of the ‘Picual’ cultivar was obtained during the 2017/2018 harvest from Castillo de Canena (Canena, Jaén, Spain). Two sample aliquots of 100 mL (see Section 2.1.1.) were frozen using the two methods explained in Section 2.1.1. and were stored at −20 °C in the dark. After 24 h of storage, the samples were thawed at room temperature and chlorophyll and pheophytin content, as well as color, were measured in duplicate (Table 1).

##### Monitoring of Chlorophyll Profile during 12 Months of Storage at −20 °C after Fast-Freezing (Study IV)

One filtered PEVOO of the ‘Picual’ cultivar was obtained during the 2018/19 harvest from Castillo de Canena (Canena, Jaén, Spain). Sample aliquots of 100 mL (see Section 2.1.1.) were frozen using the fast-freezing method explained in Section 2.1.1. The samples were stored at −20 °C in the dark for 370 days. Measurements of chlorophyll and pheophytin content and color were carried out in duplicate periodically (days 0, 33, 112, 191 and 370) (Table 1).

### 2.2. Methods

#### 2.2.1. Color Measurements

Color measurements were carried out by transmittance, using a spectrophotometer (MINOLTA CM-3500d). Light illuminant was equivalent to day light (D65) with specular component included and visual angle drifted 2° from verticality. The results were expressed as L *, a * and b * chromatic coordinates. The lightness value defines black at 0 and white at 100. The a * axis represents the green–red colors, with negative values toward green and positive values toward red. The b * axis is relative to blue–yellow colors, with negative values toward blue and positive values toward yellow.

#### 2.2.2. Analysis of Chlorophyll Profile

The extraction of pigments was carried out using a diol cartridge as described by Mateos and García-Mesa [25] with some modifications. After eluting the cartridge with 3 mL of acetone, the solvent was evaporated under a nitrogen stream in a thermoblock at 30 °C. The residue was redissolved in 0.5 mL of acetone and filtered (13 mm PTFE filters, 0.2 μm porous size). The analysis was performed by Ultra-High Performance Liquid Chromatography Diode Array Detector (UHPLC-DAD), injecting 5 μl of the extract in a Acquity-UPLC (Waters, Milford, MA, USA) equipped with an automatic injector and a Waters 2996 DAD (Waters, Milford, MA, USA). Separation was carried out using a VanGuard BEH C18 precolumn with particle size of 1.7 μm (Waters, Milford, MA, USA) and an Acquity UPLC BEH C18 column (2.1 × 50 mm and particle size of 1.7 μm) (Waters, Milford, MA, USA). Elution was performed at a flow rate of 0.6 mL/min at 30 °C, using as mobile phase methanol HPLC grade (Scharlau, Sentmenat, Spain) (solvent A) and ultrapure water (Milli-Q Millipore Corporation, Billerica, MA, USA) (solvent B). The pigments were eluted according to the following solvent gradient: from 90% (A)−10% (B), to 5% (B) at 2 min, then to 0% (B) at 6 min, and finally to 10% (B) at 8 min. Detection was carried out simultaneously at 410 nm to measure pheophytin *a*, 430 nm to measure chlorophyll *a*, 435 nm to measure pheophytin *b* and 466 to measure chlorophyll *b*. Quantification was performed using the external standard method. The chlorophyll *a* and *b* standards were purchased from Merck (Darmstadt, Germany). The pheophytin *a* and *b* standards were obtained by acidification of the chlorophyll standards as described by Sievers and Hynninen [26]. The results were given as milligrams per kilogram of olive oil.

#### 2.2.3. Statistical Analysis

To evaluate the effect of the studied storage conditions (headspace composition, storage temperature and freezing method, and storage time) on the color of the oils (study I), a three-way ANOVA (*n* = 96) was applied using SPSS Statistics (v 25, IBM, Armonk, NY, USA). *p* values lower or equal to 0.05 were considered significant. Scheffé’s test was applied in order to evaluate statistical differences between the mean values when the effect of the main factors was significant.

Pearson correlation coefficients were applied in order to assess linear correlation between color measurements and chlorophyll and pheophytin content (study I), using SPSS Statistics (v 25, IBM, Armonk, NY, USA).

## 3. Results and Discussion

### 3.1. Color Evaluation during PEVOO Storage under Different Conditions and Its Correlation with Chlorophyll and Pheophytin Content (Study I)

#### 3.1.1. Color Evaluation during PEVOO Storage under Different Conditions (Study I)

Results of the ANOVA test showed no significant effect of headspace composition on any of the chromatic coordinates (Table 2). These findings are in agreement with the results of a previous study that we performed under the same storage conditions [27]. In that study, none of the evaluated parameters (official quality parameters, phenolic compounds, volatile compounds, oxidative stability index and sensory analysis) were affected by the headspace composition, probably due to the residual oxygen remaining in the headspace of the N samples.

On the other hand, a significant effect of storage temperature on the chromatic coordinate L * was found (Table 2). The values of L *, which is an estimation of luminosity, were significantly higher in the oils stored at room temperature (RT). This is in agreement with other conservation studies, which concluded that the oils become more transparent as a consequence of the decrease in the pigment content [28,29].

A significant effect of storage time was observed on the chromatic coordinate b *, which slightly decreased over storage, showing a loss of the initial yellow color of the oils (Table 2). This could be indicating a degradation of carotenoids throughout storage, since a strong positive correlation between coordinate b * and carotenoid content has been found in previous studies [30] and would be in agreement with Gómez-Alonso et al. [31], who observed a slight decrease of carotenoid content after 21 months of storage at room temperature in the dark.

**Figure 1:**
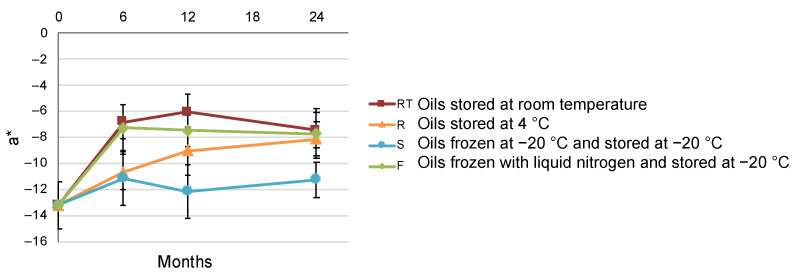
Interaction plot between the factor storage time and the factor storage temperature and freezing method for the chromatic coordinate a * (study I). Error bars correspond to the standard deviation.

Regarding chromatic coordinate a *, a significant effect of storage temperature and freezing method was observed (Table 2). Thus, comparing RT, R and S oils, a greater loss of green color (increase of coordinate a *) was observed as the storage temperature increased. These results would indicate the rate of pheophytinization of chlorophylls can be slowed down by lowering the storage temperature. The initial green color was better maintained in the S oils. Moreover, the interaction between the factor storage time and the factor storage temperature and freezing method had a significant effect on coordinate a * (Figure 1). After 24 months of storage, the S oils maintained a greener color whereas the other oils had a similar loss of green color. Regarding the effect of freezing speed, when comparing oils stored at −20 °C (S and F oils), an unexpected loss of green color was observed in the F oils (Figure 1). In fact, the evolution of a * in the F oils was very similar to that of RT oils. Since a total chlorophyll pheophytinization at 4 months of storage at RT has been reported [13,16], it could be hypothesized that a similar phenomenon occurred in the F oils. Several experiments have been carried out with the aim of clarifying this event, and their results are discussed in the subsequent sections.

#### 3.1.2. Correlation between Chlorophyll and Pheophytin Content and L * a * b * Chromatic Coordinates in Slow and Fast-Frozen PEVOO (Study I)

In order to verify whether the loss of green color observed in the F oils was due to a chlorophyll pheophytinization, correlations were established between chromatic coordinates and chlorophyll and pheophytin content in the frozen oils of study I at 12 and 24 months (*n* = 32). To this day, there are a limited number of studies in the literature correlating L * a * b * color measurements and chlorophyllic content [29,30,32,33] and this is the first time that correlations between single chlorophyll and pheophytin contents and L * a * b * color measurements are being reported. The quantification of chlorophylls in most of these previous studies was carried out spectrophotometrically by measuring the absorbance at 670 nm [29,30,32], which is due to the entire chlorophyllic fraction, that is, chlorophylls and pheophytins [11]. In these studies, results were expressed either as total chlorophyll content (i.e., chlorophylls and pheophytins) [30] or as pheophytin *a* content [29,32], since it has been assumed that pheophytin *a* is the major chlorophyllic pigment in olive oils, which might be true for stored olive oils but not necessarily so for recently extracted oils. Similarly, Cerretani et al. [33], although measuring single chlorophyll and pheophytin contents by HPLC, correlated the total chlorophyllic content with color measurements. The overlooked different chlorophyll/pheophytin ratios of the oils, which generate different colors, would explain why the results of these color-pigment correlation studies were sometimes contradictory.

Data obtained from the 32 samples of frozen PEVOO of study I corresponding to four different oils × two freezing methods × two storage times × two headspace compositions is shown in Supplementary Materials, Table S1.

In the present study, the assessment of single chlorophyll and pheophytin content versus chromatic coordinates allowed evidencing a significant negative correlation between coordinate a * and chlorophyll *a* (r = −0.676) and *b* (r = −0.489) contents (Table 3). From this result one can conclude that color measurement is a good indicator of the chlorophyll content of filtered olive oil: the higher the chlorophyll content, the lower the value of the coordinate a *. Moreover, coordinate a * showed a significant positive correlation with pheophytin *a* (r = 0.474) and *b* (r = 0.582) contents, suggesting that the observed increase of the coordinate a * in the F oils (Figure 1) was mainly due to chlorophyll pheophytinization. For instance, pheophytin average content at 12 months was much higher in the F oils (21.8 mg/kg) than in the S oils (11.2 mg/kg) (Supplementary Materials, Table S1).

### 3.2. Chlorophyll Pheophytinization in Fast-Frozen PEVOO (Studies II, III and IV)

**Figure 2:**
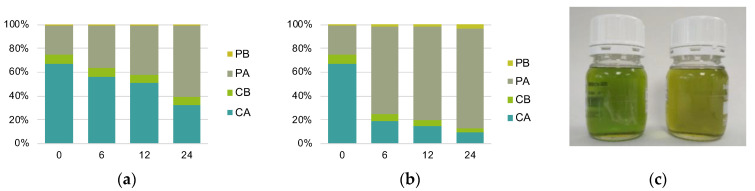
Study II: changes in the chlorophyll and pheophytin profile, expressed as % of total pigment content, in (**a**) S and (**b**) F oils during storage at −20 °C; (**c**) Image of the S (left) and F (right) oils after 6 months of storage at −20 °C. Abbreviations: S, oil frozen at −20 °C; F, oil frozen with liquid nitrogen; CA, chlorophyll *a*; CB, chlorophyll *b*; PA, pheophytin *a*; PB, pheophytin *b*.

To confirm the hypothesis that the loss of green color in the F oils was due to chlorophyll pheophytinization, a second experiment was carried out: chlorophyll and pheophytin content and color of a PEVOO frozen with two freezing speeds (S and F) were monitored during 24 months of storage at −20 °C (study II). Color results showed the same trend observed in study I. Regarding chlorophyllic pigments, results depicted in Figure 2a,b show that, as hypothesized, there was more pheophytinization in the F oil than in the S oil. The pheophytinization was mainly at the expense of the *a* form: at the end of the study, chlorophyll *a* decreased a 62.5% in the S oil, while in the F oil the reduction was a 89%. In addition, pheophytinization in the F oil occurred at the beginning of the study: chlorophyll *a* content was reduced by 69.5% after the first 6 months of storage, which translated into a browner color compared to the S oil, which had a greener color (Figure 2c).

As stated previously, pheophytinization is the result of a replacement of the magnesium ion for 2 hydrogen atoms. This loss of the magnesium ion can be originated by heat, low pH and, presumably, by the action of non-identified substances with magnesium dechelating activity [16,34]. A previous study concluded that pheophytinization reactions taking place during storage of virgin olive oils were not interrelated with the free acidity of the oils, hypothetically attributing them to the presence of these Mg-dechelating substances that may be released during fruit milling and paste beating and transferred to the oils [13]. Even if that were the case, the low rate of pheophytinization observed in the S oils indicates that if those Mg-dechelating substances were present in the oils, their activity is reduced by storage at −20 °C. With results of study II, this pheophytinization appeared to have taken place at the moment of freezing with liquid nitrogen and we hypothesized that the sudden drop of temperature and the speed of freezing could have originated this loss of the magnesium ion.

**Figure 3:**
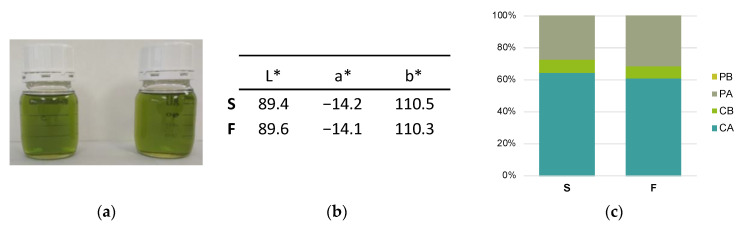
Results of the 24 h test (study III): (**a**) Image of the S (left) and F (right) oils after 24 h at −20 °C; (**b**) Color measurements; (**c**) Chlorophyll and pheophytin profile of S and F oils, expressed as %. Abbreviations: S, oil frozen at −20 °C; F, oil frozen with liquid nitrogen; CA, chlorophyll *a*; CB, chlorophyll *b*; PA, pheophytin *a*; PB, pheophytin *b*.

In order to ascertain whether pheophytinization occurred at the moment of freezing with liquid nitrogen, a third experiment was carried out: two samples of the same PEVOO were frozen at the two freezing speeds (S and F) and their pigment profile and color were measured after 24 h of storage at −20 °C (study III). Results showed no difference in chlorophyll and pheophytin content and color between the S and F oil after 24 h of storage at −20 °C (Figure 3), indicating that pheophytinization did not take place at the moment of freezing with liquid nitrogen. Considering that in study II pheophytinization in the F oil occurred in the first 6 months of storage at −20 °C (Figure 2), the results suggested that this reaction is the consequence of the combined effect of fast-freezing and storing at −20 °C.

**Figure 4:**
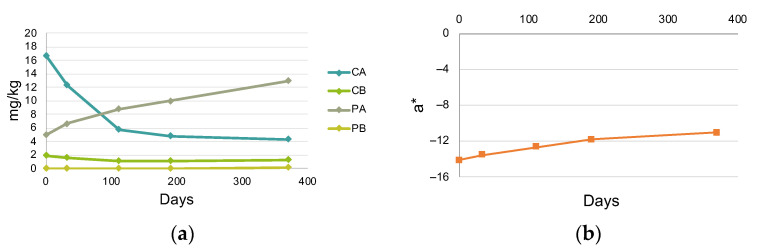
Changes in (**a**) the chlorophyll and pheophytin profile and (**b**) chromatic coordinate a * during storage at –20 °C of an oil frozen with liquid nitrogen (study IV). Abbreviations: CA, chlorophyll *a*; CB, chlorophyll *b*; PA, pheophytin *a*; PB, pheophytin *b*.

The monitoring of chlorophyll, pheophytin and color during 12 months of storage at −20 °C (study IV) confirmed that a loss of chlorophyll *a* (Figure 4a), accompanied by a loss of green color (Figure 4b) occurs in fast-frozen PEVOO. The decrease of chlorophyll *a* was partly due to pheophytinization and took place mostly during the first 112 days of storage at −20 °C (Figure 4b).

In summary, freezing olive oils with a high chlorophyll content with liquid nitrogen and storing them at −20 °C originated a more rapid degradation of chlorophyll *a* compared to freezing at −20 °C. This degradation, partly caused by pheophytinization, occurred largely during the first 112 days of storage.

## 4. Conclusions

From the results of study I we can conclude that freezing and storing at −20 °C slows down the rate of pheophytinization of chlorophylls in PEVOO, thus effectively preserving their initial chlorophyll profile, which is responsible of their highly appreciated green color. Contrarily, storage at room temperature and at 4 °C entailed a significant loss of green color after 6 and 12 months, respectively. On the other hand, results of studies I, II, III and IV show that freezing with liquid nitrogen and storing at −20 °C involves a rapid degradation of chlorophyll a, mainly due to pheophytinization, during the first 112 days of storage. The mechanism by which this degradation occurs remains unclear. Thus, more studies are needed to fully understand how this event takes place. Lastly, our findings show that the increase in chromatic coordinate a * is a good indicator of chlorophyll pheophytinization in olive oil.

## Figures and Tables

**Table 1:** Experimental designs of studies I, II, III and IV.

Study I (Factorial Design)						
Determinations: color parameters						
Time (months)	0	6	12	24		
Oils	4	4	4	4		
Headspace composition	O	O/N	O/N	O/N		
Temperature and freezing method	RT	RT/R/S/F	RT/R/S/F	RT/R/S/F		
*n*	4	32	32	32		100
Determinations: chlorophyll and pheophytin content						
Time (months)			12	24		
Oils			4	4		
Headspace composition			O/N	O/N		
Temperature and freezing method			S/F	S/F		
*n*			16	16		32
**Study II (Factorial Design)**						
Determinations: color parameters and chlorophyll and pheophytin content						
Time (months)	0	6	12	24		
Oils	1	1	1	1		
Headspace composition	O	O/N	O/N	O/N		
Temperature and freezing method		S/F	S/F	S/F		
*n*	1	4	4	4		13
**Study III**						
Determinations: color parameters and chlorophyll and pheophytin content						
Time (hours)	24					
Oils	1					
Headspace composition	O					
Temperature and freezing method	S/F					
*n*	2					2
**Study IV**						
Determinations: color parameters and chlorophyll and pheophytin content						
Time (days)	0	33	112	191	370	
Oils	1	1	1	1	1	
Headspace composition	O	O	O	O	O	
Temperature and freezing method	F	F	F	F	F	
*n*	1	1	1	1	1	5

O, air; N, nitrogen; RT, room temperature; R, 4 °C; S, oils frozen at −20 °C and stored at −20 °C; F oils frozen with liquid nitrogen and stored at −20 °C.

**Table 2:** Effect of headspace composition, storage temperature and freezing method and storage time on the chromatic coordinates L * a * b * (study I).

		Headspace Composition			Temperature and Freezing Method					Time (Months)			
	Fresh ^1^	O ^2^	N ^2^	s.e.	RT ^2^	R ^2^	S ^2^	F ^2^	s.e.	6 ^2^	12 ^2^	24 ^2^	s.e.
L * ^3^	86.6	88.7	88.7	0.281	90.5 ^a^	88.5 ^b^	87.4 ^b^	88.3 ^b^	0.397	88.3	88.5	89.3	0.344
a *	−13.2	−8.6	−8.9	0.237	−6.8 ^a^	−9.3 ^b^	−11.5 ^c^	−7.5 ^a^	0.335	−9	−8.7	−8.6	0.29
b *	122.2	120.3	120.6	0.673	120.3	121.3	121.3	118.7	0.951	121.7 ^a^	121.5 ^a^	118.1 ^b^	0.824

^1^ Mean values of the four oils. ^2^ Means from the three-way ANOVA (*n* = 96 corresponding to two headspace compositions × four temperature and freezing methods × three storage times × four different oils). ^3^ The means within each row for each factor, labelled by different letters, are significantly different (*p* ≤ 0.05). The interaction between time and temperature and freezing method is statistically significant (*p* ≤ 0.05) for the chromatic coordinate a *. s.e., standard error; O, air; N, nitrogen; RT, room temperature; R, 4 °C; S, oils frozen at −20 °C and stored at −20 °C; F, oils frozen with liquid nitrogen and stored at −20 °C.

**Table 3:** Pearson correlation coefficients (r) between chlorophyll and pheophytin content and the chromatic coordinates L * a * b * in frozen Premium extra virgin olive oil (PEVOO) (study I).

	L *	a *	b *
CA	−0.286	−0.676 **	−0.013
CB	−0.525 **	−0.489 **	0.109
PA	−0.148	0.474 **	−0.231
PB	0.090	0.582 **	−0.130

** *p* ≤ 0.01; CA, chlorophyll *a*; CB, chlorophyll *b*; PA, pheophytin *a*; PB, pheophytin *b*.

## Data Availability

The datasets generated for this study are available on request to the corresponding author.

## Supplementary Materials

The following supporting information can be downloaded at: https://www.mdpi.com/article/10.3390/foods12010222/s1. Table S1: Data matrix used for the calculations of Pearson correlation coefficients between chlorophyll and pheophytin content (mg/kg) and chromatic coordinates (L * a * b *) of four frozen PEVOO (study I, *n* = 32).
