# Supplementary material for: Effect of the Storage Conditions and Freezing Speed on the Color and Chlorophyll Profile of Premium Extra Virgin Olive Oils
Source: Foods. 2023 Jan 3;12(1):222. doi: 10.3390/foods12010222 (PMC9819069; doi:10.3390/foods12010222)
Supplement: Supplementary file 1 [file foods-12-00222-s001.zip › foods-2068115-supplementary.pdf]

Supplementary Materials

Effect of the Storage Conditions and Freezing Speed on the Color and Chlorophyll Profile of Premium Extra Virgin Olive Oils

Anna Díez-Betriu, Julen Bustamante, Agustí Romero, Antonia Ninot, Alba Tres, Stefania Vichi* and Francesc Guardiola.

* Correspondence: stefaniavichi@ub.edu

Table S1. Data matrix used for the calculations of Pearson correlation coefficients between chlorophyll and pheophytin content (mg/kg) and chromatic coordinates (L_*a_*_b*) of 4 frozen premium EVOO (study I, n=32).

Oil	Month	Freezing speed	Headspace composition	CA	CB	PA	PB	L_*	a_*	b_*
EP	12	S	O	14.48	2.20	15.33	0.09	88.3	–-16.2	108.9
EP	12	S	N	18.44	2.38	11.66	0.07	88.5	-–15.6	109.2
EP	12	F	O	5.63	1.82	24.28	0.33	89.9	-–13.5	109.6
EP	12	F	N	3.90	1.32	25.36	0.75	89.8	-–13.7	109.9
EP	24	S	O	7.47	1.46	12.23	0.05	89.8	-–14.4	109.1
EP	24	S	N	6.57	1.43	14.04	nd	89.4	-–12.2	108.0
EP	24	F	O	2.39	0.80	17.65	0.67	90.3	-–11.4	105.0
EP	24	F	N	1.75	0.66	18.71	0.64	89.8	-–11.1	105.3
LP	12	S	O	9.44	1.36	9.77	0.08	87.7	-–12.4	117.5

Oil	Month	Freezing speed	Headspace composition	CA	CB	PA	PB	L*	a*	b*
LP	12	S	N	8.02	1.27	10.92	0.11	87.6	−13.5	118.2
LP	12	F	O	0.80	1.00	21.86	1.17	88.1	−8.6	116.9
LP	12	F	N	0.62	0.44	27.10	2.16	88.9	−8.2	112.9
LP	24	S	O	12.77	2.33	13.22	0.06	87.5	−13.3	117.7
LP	24	S	N	13.07	2.49	14.80	0.10	87.8	−10.9	116.3
LP	24	F	O	0.73	0.57	28.24	1.96	89.1	−9.3	108.9
LP	24	F	N	1.26	0.68	27.63	1.77	88.9	−9.5	111.0
EA	12	S	O	7.43	2.03	14.93	0.21	85.3	−8.4	125.4
EA	12	S	N	9.88	2.21	13.34	0.07	85.0	−10.0	124.8
EA	12	F	O	0.45	0.77	22.28	1.14	86.0	−4.8	125.6
EA	12	F	N	5.31	1.88	17.12	0.14	85.5	−6.8	125.9
EA	24	S	O	10.95	2.30	16.02	0.08	85.2	−9.2	125.2
EA	24	S	N	12.65	2.29	13.66	0.13	85.1	−9.9	124.9
EA	24	F	O	0.39	0.88	27.61	1.30	85.9	−4.8	125.7
EA	24	F	N	0.37	0.61	27.29	1.60	86.1	−4.7	125.3
LA	12	S	O	8.85	1.21	6.27	0.17	89.9	−12.3	123.7
LA	12	S	N	7.90	1.26	6.33	0.19	89.9	−11.9	123.1
LA	12	F	O	0.13	0.24	16.20	1.08	90.7	−7.8	119.4
LA	12	F	N	1.05	0.79	12.88	0.34	90.5	−8.2	120.7
LA	24	S	O	7.07	1.28	9.01	0.05	90.0	−12.2	122.6

Oil	Month	Freezing speed	Headspace composition	CA	CB	PA	PB	L*	a*	b*
LA	24	S	N	9.09	1.29	7.13	0.04	90.1	–10.9	121.3
LA	24	F	O	0.11	0.19	16.57	0.95	91.0	–8.0	118.2
LA	24	F	N	0.42	0.27	16.11	0.84	90.9	–8.0	118.5

Abbreviations: nd, not detected; EP, premium EVOO from the Picual cultivar produced at the beginning of the harvest; LP, premium EVOO from the Picual cultivar produced at the end of the harvest; EA, premium EVOO from the Arbequina cultivar produced at the beginning of the harvest; LA, premium EVOO from the Arbequina cultivar produced at the end of the harvest; S, oils frozen at –20 °C and stored at –20 °C; F oils frozen with liquid nitrogen and stored at –20 °C; O, air; N, nitrogen; CA: chlorophyll *a*; CB: chlorophyll *b*; PA: pheophytin *a*; PB: pheophytin *b*.
